# Phylogenetic Analysis of HIV-1 Genomes Based on the Position-Weighted K-mers Method

**DOI:** 10.3390/e22020255

**Published:** 2020-02-23

**Authors:** Yuanlin Ma, Zuguo Yu, Runbin Tang, Xianhua Xie, Guosheng Han, Vo V. Anh

**Affiliations:** 1 Hunan Key Laboratory for Computation and Simulation in Science and Engineering and Key Laboratory of Intelligent Computing and Information Processing of Ministry of Education, Xiangtan University, Xiangtan 411105, China; 201590110068@smail.xtu.edu.cn (Y.M.); 201831510085@smail.xtu.edu.cn (R.T.); hangs@xtu.edu.cn (G.H.); vanh@swin.edu.au (V.V.A.); 2 School of Economics, Zhengzhou University of Aeronautics, Zhengzhou 450046, China; 3 School of Electrical Engineering and Computer Science, Queensland University of Technology, GPO Box 2434, Brisbane, QLD 4001, Australia; 4 School of Mathematical and Computer Science, Gannan Normal University, Ganzhou 341000, China; xiexianhua@gnnu.edu.cn; 5 Faculty of Science, Engineering and Technology, Swinburne University of Technology, P.O. Box 218, Hawthorn, VIC 3122, Australia

**Keywords:** Alignment-free, HIV-1 virus, phylogenetic analysis, position-weighted *k*-mers, Robinson–Foulds distance

## Abstract

HIV-1 viruses, which are predominant in the family of HIV viruses, have strong pathogenicity and infectivity. They can evolve into many different variants in a very short time. In this study, we propose a new and effective alignment-free method for the phylogenetic analysis of HIV-1 viruses using complete genome sequences. Our method combines the position distribution information and the counts of the *k*-mers together. We also propose a metric to determine the optimal *k* value. We name our method the *Position-Weighted k-mers* (*PWkmer*) method. Validation and comparison with the Robinson–Foulds distance method and the modified bootstrap method on a benchmark dataset show that our method is reliable for the phylogenetic analysis of HIV-1 viruses. *PWkmer* can resolve within-group variations for different known subtypes of Group M of HIV-1 viruses. This method is simple and computationally fast for whole genome phylogenetic analysis.

## 1. Introduction

Human Immunodeficiency Viruses (HIVs) are retroviruses which are the causative agents of the global pandemic of Acquired Immunodeficiency Syndrome (AIDS) [1]. There are two types of HIVs: Type 1 (HIV-1 viruses) and Type 2 (HIV-2 viruses). HIV-1 viruses are known to originate from the Simian Immunodeficiency Viruses (SIVs) found in central and eastern African chimpanzees, which form the most common pathogenic strain of HIV viruses and have a high mortality rate [2]. Usually, HIV-1 viruses are divided into a major group (Group M) and two or more minor groups, namely Groups N, O, and possibly Group P. Group M is further divided into subtypes A, B, C, D, E, F, J, K. The subtypes A and F are further divided into sub-subtypes (A1, A2) and (F1, F2) based on differential phylogenetic clustering, respectively. Two or more HIV-1 subtypes can recombine and form Circulating Recombinant Forms (CRFs) [3]. Obviously, classification of HIV-1 strains into subtypes, sub-subtypes, and CRFs is a complex issue, which leads to major problems in the development of vaccines against HIV-1. These problems include high genetic variation, the fast evolution of different variants, and sequence diversity. The first task to solve these problems is how to obtain the phylogenetic relationships of HIV-1 genomes quickly and accurately. Traditional HIV-1 phylogenetic analysis methods are based on multiple sequence alignment. Although alignment-based methods generally yield excellent results when the sequences are closely related and can be reliably aligned, there are two limitations. Firstly, they lead to conflicting results by using different genes or genome fragments. Secondly, alignment-based methods are generally time-consuming and have high computational complexity when they are directly applied to whole-genome comparisons and phylogenetic studies [4]. Therefore, several alignment-free methods have been developed to overcome the critical limitations of alignment [5,6,7,8,9,10,11,12,13,14]. In particularly, several alignment-free methods for HIV genome comparison have been developed in the past few decades. For example, Wu et al. [5] used the complete composition vector representation proposed by Hao and Qi [15] for the phylogenetic analysis of HIV-1 genomes, and obtained some acceptable results. Pandit et al. [16] used multifractal measures to capture the genomic variation in the different retroviral species. However, this multifractal method cannot resolve the subtle variations in the subtypes of Group M of HIV-1 viruses. The first usage of *k*-mers (substring of length *k*) counts for biological sequence comparison was implemented by Blaisdell [17]. Subsequently, a lot of alignment-free methods using *k*-mers emerged. Yang and Wang [7] proposed a novel statistical measure for sequence comparison on the basis of *k*-mers counts, which removes the influence of the length of sequences, and obtained some acceptable results for the phylogenetic analysis of HIV-1 genomes. Chang et al. [8] proposed a cumulative Markov mutual information (CMMI) method which was derived from several *k*-mers distributions in different genome sequences, and reported some computational results on the HIV-1 subtyping. These results are slightly different from those reported in the NCBI (National Center for Biotechnology Information Search database). In addition, there are other alignment-free methods that may also be used for HIV-1 genome comparisons, such as the gene content-based method [18], the data compression method [19], the fractal method [20], the CVTree method [21], the inter-amino-acid distance method [10], the higher-order Markov model [11], the dynamical language model [6,12], a method using spaced-word frequencies [9], and a method based on the distribution of *k*-mer intervals [22]. All these alignment-free methods for comparing biological sequences are intended to extract hidden information from the whole genomes, but from different angles.

In this study, we present a new alignment-free method based on position-weighted *k*-mers to capture the subtle variations from the complete genome sequences of HIV-1 viruses. In our method, the effects of *k*-mers counts and *k*-mers position distributions are combined to capture more evolutionary information. On the basis of the proposed method, we report and discuss the results on the HIV-1 subtyping. More importantly, the resulting phylogenetic tree of 44 HIV genome sequences is quite consistent with the accepted taxonomy from NCBI. Our results show that the new method works as well as the conventional alignment-based phylogenetic methods and other alignment-free methods, but is simpler and requires much less computational time and resources. Moreover, our approach can be applied to study the subtype clustering and phylogenetic relationships of a large volume of genome sequences. The source codes of our method can be downloaded from https://github.com/myl446/HivStudy. The detailed information please see the Supplementary Materials.

## 2. Materials and Methods

### 2.1. Complete Genome Datasets

Twenty of the 21 genomes used in Chang et al. [8] are included in the 43 genomes used in Wu et al. [5]. For the phylogenetic analysis of HIV-1 complete genomes, we used a dataset which is composed of 44 HIV complete genomes (43 HIV complete genomic sequences used in the literature [5] and a misplaced sequence of the article categorization [8]). This dataset includes the subtypes A, B, C, D, F, G, J, K, H of the HIV-1 Groups M, O, and N, and a CPZ sequence. All of these sequences can be downloaded from the Los Alamos National Laboratory HIV Sequence Database (http://www.hiv.lanl.gov/). Specific accession, subtype, length (bp), and area are listed in Table 1. Many studies suggested that all of the translated protein amino acid sequences from the genome is a better choice than whole genome DNA sequences and coding parts of complete genomes for genome-based phylogeny reconstruction [6,12,21,23]. However, after computational comparisons and theoretical analysis, we found that our present method is only suitable for whole genome DNA sequences.

### 2.2. The Measure of Position-Weighted K-mers

Assume that $s_{1}s_{2}\ldots s_{k}$ is a *k*-mer, where $s_{i}\in \left\{A,T,C,G\right\}$. If the *k*-mer $s_{1}s_{2}\ldots s_{k}$ occurs in a given nucleic acid sequence *X*, then we denote by $P_{s_{1}s_{2}\ldots s_{k}}$ the vector composed of the positions of $s_{1}s_{2}\ldots s_{k}$ in *X* and by $P_{s_{1}s_{2}\ldots s_{k}}\left(i\right)$ its ith element. If $s_{1}s_{2}\ldots s_{k}$ does not exist in *X*, $P_{s_{1}s_{2}\ldots s_{k}}$ is a zero vector. For example, we consider the 2-mers position vectors for the following short nucleic acid sequence of length 20: X=TAAGCCGCATTAGCTGGTTT. We get $P_{AA}=\left(2\right),P_{GA}=\left(0\right),P_{GC}=\left(4,7,13\right)\ldots$. These *k*-mers position vectors can effectively capture the distribution information of each *k*-mer in the given sequence. For a fixed *k*, we can reverse this sequence by some *k*-mers position vector. Furthermore, if a *k*-mer exists in the given sequence, the counts of this *k*-mer in the nucleic acid sequence are equal to the length of its corresponding position vector. Therefore, we can use the following 2-mers position vector to reconstruct the nucleic acid sequence used in this example:

$$\begin{array}{cc} \; & P_{AA}=\left(2\right),P_{AC}=\left(0\right),P_{AG}=\left(3,12\right),P_{AT}=\left(9\right),P_{CA}=\left(0\right),P_{CC}=\left(5\right),\\ \; & P_{CG}=\left(6\right),P_{CT}=\left(14\right),P_{GA}=\left(0\right),P_{GC}=\left(4,7,13\right),P_{GG}=\left(16\right),\\ \; & P_{GT}=\left(17\right),P_{TA}=\left(1,11\right),P_{TC}=\left(0\right),P_{TG}=\left(15\right),P_{TT}=\left(10,18,19\right). \end{array}$$

The 2-mers AC,CA,GA, and TC do not appear in this example. Now, we reverse the given nucleic acid sequence as follows:

$$\begin{array}{ll} P_{TA}=\left(1,11\right) & TA\ldots TA\ldots \\ P_{AA}=\left(2\right) & TAA\ldots TA\ldots \\ P_{AG}=\left(3,12\right) & TAAG\ldots TA.AG\ldots \\ P_{GC}=\left(4,7,13\right) & TAAGC.GCTA.AGC\ldots \\ P_{CG}=\left(6\right) & TAAGCGGCTA.AGC\ldots \\ P_{TA}=\left(1,11\right) & TAAGCGGCTATAGC\ldots \\ P_{TG}=\left(15\right) & TAAGCGGCTATAGCTG\ldots \\ P_{GT}=\left(17\right) & TAAGCGGCTATAGCTGGT\ldots \\ P_{TT}=\left(10,18,19\right) & TAAGCGGCTATAGCTGGTTT. \end{array}$$

Suppose

$$P_{s_{1}s_{2}\ldots s_{k}}=\left(p_{1},p_{2},\ldots ,p_{m}\right),$$

where *m* is the count of $s_{1}s_{2}\ldots s_{k}$ in the given nucleic acid sequence. The measure of $s_{1}s_{2}\ldots s_{k}$ based on its position in the sequence, denoted $f(s_{1}s_{2}\ldots s_{k})$, is defined as

$$f\left(s_{1}s_{2}\ldots s_{k}\right)=\left\{\begin{array}{ll} \frac{\left(\frac{p_{1}}{L}+\frac{p_{2}}{L}+\cdots +\frac{p_{m}}{L}\right)}{L-k+1}, & m\neq 0,\\ 0, & m=0, \end{array}\right.$$

where *L* is the length of the given sequence.

After simplifying, the following form is obtained:

(1) $$f\left(s_{1}s_{2}\ldots s_{k}\right)=\left\{\begin{array}{ll} \frac{\sum_{i=1}^{m}p_{i}}{(L-k+1)L}, & m\neq 0,\\ 0, & m=0. \end{array}\right.$$

To calculate the similarity distances between different sequences, we should assign a measure to each *k*-mer based on the *k*-mers position information. In this study, we use Formula (1) to extract evolutionary information from the nucleic acid sequence. As compared with the other *k*-mers-based methods, our method involves not only the counts of $s_{1}s_{2}\ldots s_{k}$, but also all the occurring positions of $s_{1}s_{2}\ldots s_{k}$. The method proposed here combines the position distribution information and the counts of the *k*-mers together, which can capture more phylogenetic information from sequences. For example, for two sequences $X_{1}=CCAGTTGCCC,X_{2}=CCCAGTTGCC$, the counts of CC in $X_{1}$ and $X_{2}$ are both 3. If we only consider the frequency of $CC,N_{X_{1}}\left(CC\right)=N_{X_{2}}\left(CC\right)$, the phylogenetic information of CC captured by N(CC) is not sufficient. However, when we use our measure $f\left(s_{1}s_{2}\ldots s_{k}\right),f_{X_{1}}\left(CC\right)=0.2,f_{X_{2}}\left(CC\right)=0.133$. Hence, more phylogenetic information of CC can be captured by f(CC).

### 2.3. Distance Calculations

There are a total of $4^{k}$ distinct *k*-mers for a fixed *k*. Sorting these *k*-mers in a fixed order, we can obtain a $4^{k}$-dimensional feature representation vector denoted by $\left(S_{1},S_{2},\ldots ,S_{4^{k}}\right)$. Then, according to the feature vector and our measure for *k*-mers, we obtain the corresponding vector $\left(f_{1},f_{2},\ldots ,f_{4^{k}}\right)$. For given *n* nucleic acid sequences, we can get a $n\times 4^{k}$ feature matrix *F* ($f_{i,j}$ represents the jth feature of the sequence *i*, i=1,2,…,n, $j=1,2,\ldots ,4^{k}$, *k* is the length of *k*-mers):

(2) $$\left[\begin{array}{cccc} f_{1,1} & f_{1,2} & \ldots & f_{1,4^{k}}\\ f_{2,1} & f_{2,2} & \ldots & f_{2,4^{k}}\\ \vdots & \vdots & \ddots & \vdots \\ f_{n,1} & f_{n,2} & \ldots & f_{n,4^{k}} \end{array}\right]$$

There are many methods to calculate the distance between two vectors. In this paper, we use the Manhattan distance [24,25], which was commonly used to analyze similarity of biological sequences. Assuming that $Y=(f_{Y_{1}},f_{Y_{2}},\ldots ,f_{Y_{4^{k}}})$ and $Z=(f_{Z_{1}},f_{Z_{2}},\ldots ,f_{Z_{4^{k}}})$ represent the feature vectors of the two sequences calculated by our method, we use the following formula to calculate the Manhattan distance:

(3) $$d\left(Y,Z\right)=\overset{4^{k}}{\underset{l=1}{\sum }}\left|f_{Y_{l}}-f_{Z_{l}}\right|.$$

For the experimental dataset, we can obtain the pairwise distance matrix based on the Manhattan distance. The distance matrix can depict the similarity information of the nucleic acid sequences. After generating the distance matrix, we use it as an input to the MEGA7 [26] and use the Neighbor-Joining (NJ) program [27] to generate the phylogenetic tree. We name this method the *Position-Weighted k-mers (PWkmer)* method.

### 2.4. Selection of the *k* Value

The *k* value in our *PWkmer* method is very important to capture the subtle variation information of a genome sequence. Certainly, a larger value of *k* will give a vector containing finer evolutionary information. However, many *k*-mers with large value of *k* will not occur in the genome sequence. At the same time, some important information may be discarded and noise will dominate when a large value of *k* is considered. In order to determine the optimal *k* value, similar to the definition of the matrix in Shannon entropy by Zhao et al. [28], we consider a scoring scheme score(k) to estimate the distribution of *k*-mers defined as

(4) $$score\left(k\right)=-\frac{1}{n}\overset{n}{\underset{i=1}{\sum }}\overset{4^{k}}{\underset{j=1}{\sum }}f_{ij}\log f_{ij}.$$

Note that the larger score(k) is, the more information can be extracted by the *k*-mers distribution.

**Figure 1:**
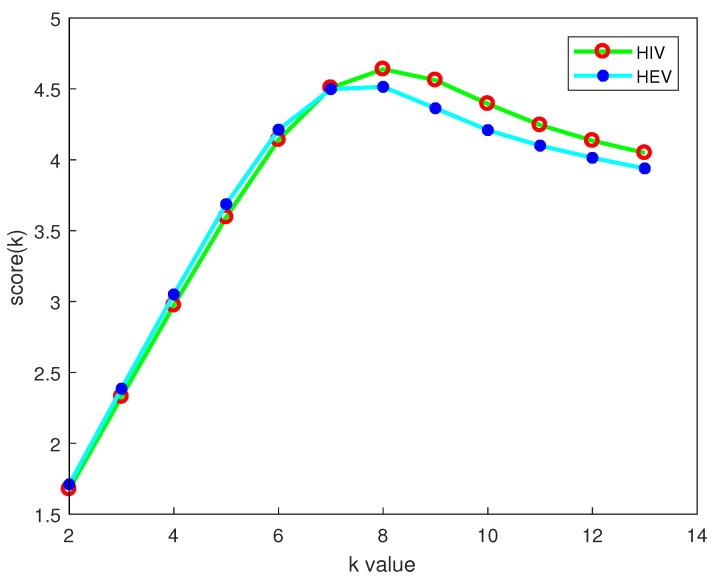
The trend chart of *k* value vs. scoring scheme *score(k)*. The red circles represent the score of the HIV dataset for different *k* values, and the blue dots represent the score of the HEV dataset for different *k* value.

The relation between some score(k) and *k* in our experiment using the dataset of HIV is given in Figure 1. It can be seen that score(k) reaches the largest value at k=8 and decreases after k>8. This indicates that the difference between these genome sequences in 9-mers distributions is decreasing. At the same time, it will require a lot of memory to be computationally efficient when *k* increases. Therefore, we determine k=8 as the optimal value in our *PWkmer* method to distinguish these genome sequences.

### 2.5. Accuracy Test of the Phylogenetic Tree Based on the Robinson–Foulds Distance and Robustness Test Using the Modified Bootstrap Method

There are many methods to evaluate the accuracy of tree reconstruction methods. The Robinson–Foulds [29] metric is a way to measure the distance between unrooted phylogenetic trees. In this work, we use it to evaluate the accuracy of the trees we constructed. In general, subtyping of virus species is usually based on multiple sequence alignment in the field of virology. Therefore, we firstly find the reference tree of the species studied. Then, the Robinson–Foulds distance between our tree and the reference tree is implemented in the treedist program of the Phylip package [30]. The smaller the Robinson–Foulds distance is, the more accurate our tree is.

We also use the modified version of the bootstrap method proposed by Yu et al. [6] to evaluate the robustness of the trees we constructed. The workflow is as follows: first, we construct Matrix (2) with each row being the feature vector of each genome sequence. Second, we resample with repeats the $4^{k}$ columns to construct a new matrix. Third, we compute the Manhattan distances between any two row vectors based on the new matrix. Then, a distance matrix can be obtained based on the resampled matrix. Fourth, the same tree-building method is used to rebuild the tree. Finally, we repeat the above process a large number of times (usually 100 times). The frequency with which a particular phylogenetic branch emerges can be used as a measure of its reliability.

## 3. Results

### 3.1. Subtyping of HIV-1 Based on PWkmer Feature for Complete Genome Sequences

**Figure 2:**
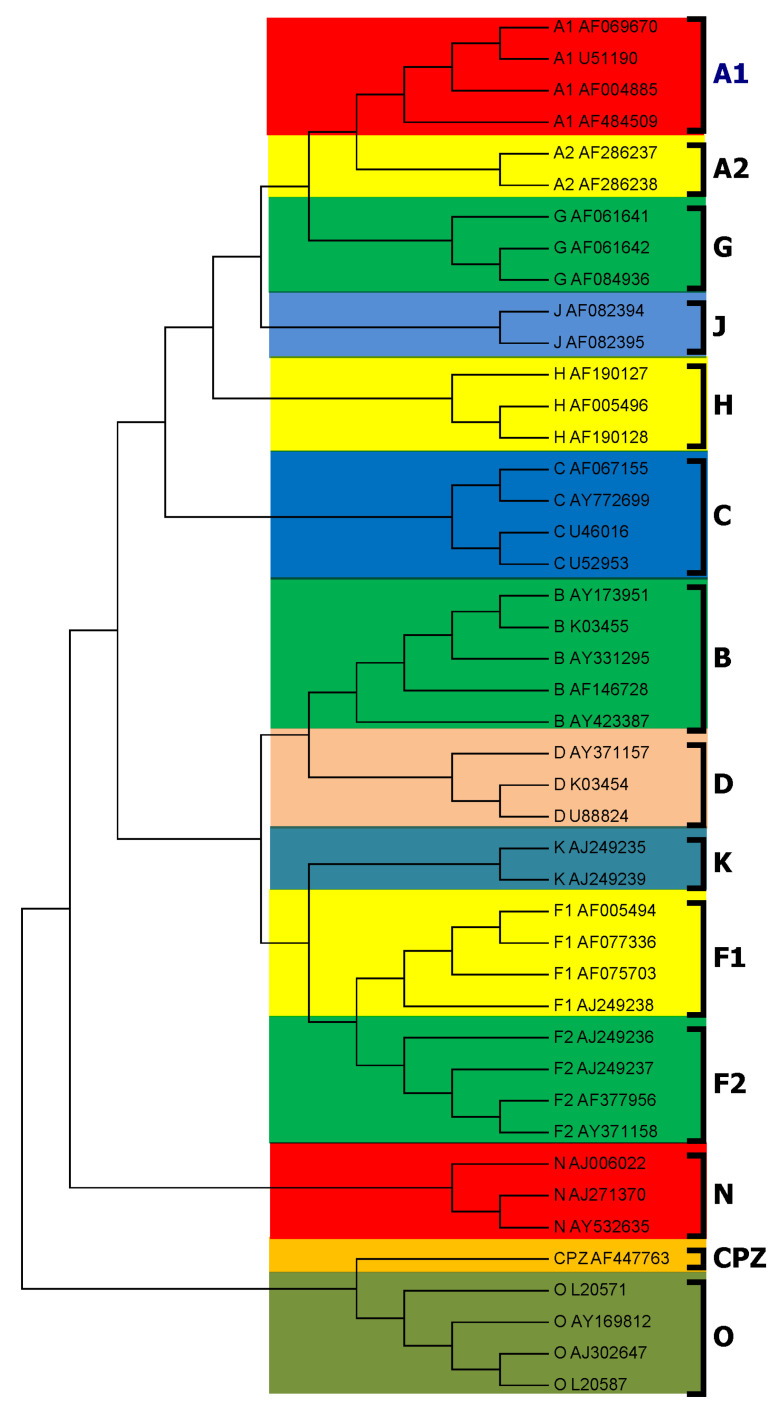
Subtyping of HIV based on position weighted *k*-mers feature for whole genome sequences. The Neighbor-Joining (NJ) tree of 44 HIV whole genomes is constructed by position weighted *k*-mers feature distance matrix (k=8).

Using our *PWkmer* method, the phylogenetic analysis was performed on 44 HIV complete genome sequences listed in Table 1. We reconstructed the phylogenetic trees for k=2,3,…,10. The phylogenetic tree for k=8 is the best among these trees, which agree with our theoretical optimal value for *k*. The obtained phylogenetic tree for k=8 is shown in Figure 2. It is seen in Figure 2 that the strains from the same subtype are closely clustered together. Forty-four HIV genomes are distinctly divided into four groups: Group M is the main group of viruses in the HIV-1 global pandemic, and it contains multiple subtypes (A, B, C, D, F, G, H, J, K). Groups N and O are very distinctive forms of the viruses, which originate from other primates and then infect human beings. Group CPZ contains the closest non-human primate viruses related to HIV-1, which are the primate viruses isolated from chimpanzees. In this tree, all subtypes are clearly grouped together as distinct branches, and the closeness relationships among the subtypes are also well demonstrated. Namely, Subtypes B and D are closer to each other than to the others, and Subtype F(A) indeed contains two distinguishable Sub-subtypes F1 and F2 (A1 and A2). All these results are in very good agreement with those of previous studies [5,31].

**Figure 3:**
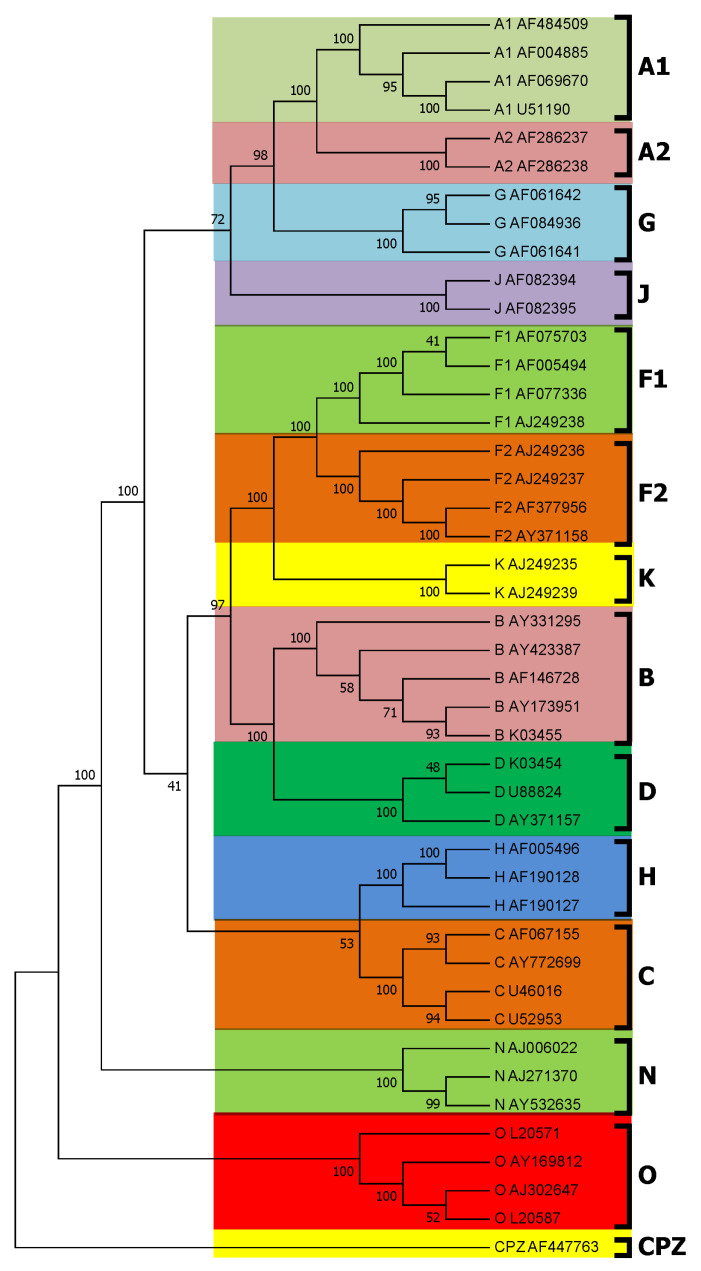
Subtyping of HIV based on alignment for whole genome sequences. The NJ tree of 44 HIV whole genomes is constructed by ClustalX.

**Figure 4:**
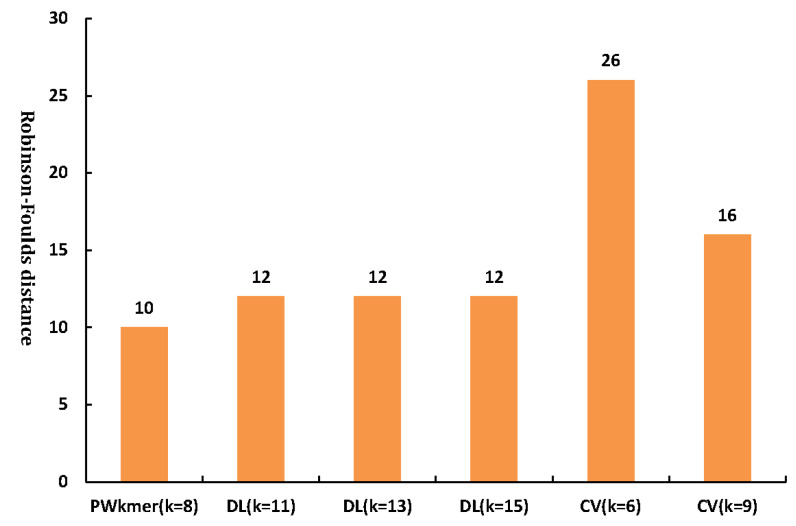
Robinson–Foulds distance between phylogenetic trees reconstructed by the *PWkmer* method, the CVTree method [20], the DLTree [12] method, and the tree reconstructed by ClustalX method for 44 HIV genome sequence in Table 1 (we selected their optimal result tree by CVTree and DLTree).

To verify the accuracy and reliability of the tree constructed by the *PWkmers* method, we used ClustalX [32], which is a multiple sequence alignment program, to construct a reference tree of 44 HIV complete genome sequences. As shown in Figure 3, this tree is quite consistent with the accepted taxonomy from NCBI. Moreover, we calculated the Robinson–Foulds distance between the tree constructed by the *PWkmer* method and the tree constructed by ClustalX. DLTree [12] and CVTree [21] are the more classical alignment-free methods in the publicized existing software of phylogenetic analysis. We also used them to construct the phylogenetic trees for 44 HIV complete genome sequences. At the same time, we computed the Robinson–Foulds distance between these trees constructed by the *PWkmers* method, CVTree [21], DLTree [12], and the tree constructed by ClustalX for 44 HIV complete genome sequences. The distances of the tree constructed by each method to the tree constructed by ClustalX are shown in Figure 4. The Robinson–Foulds distance of the *PWkmers* method is minimal, which illustrates that our results are the most closely consistent with the results of ClustalX.

**Figure 5:**
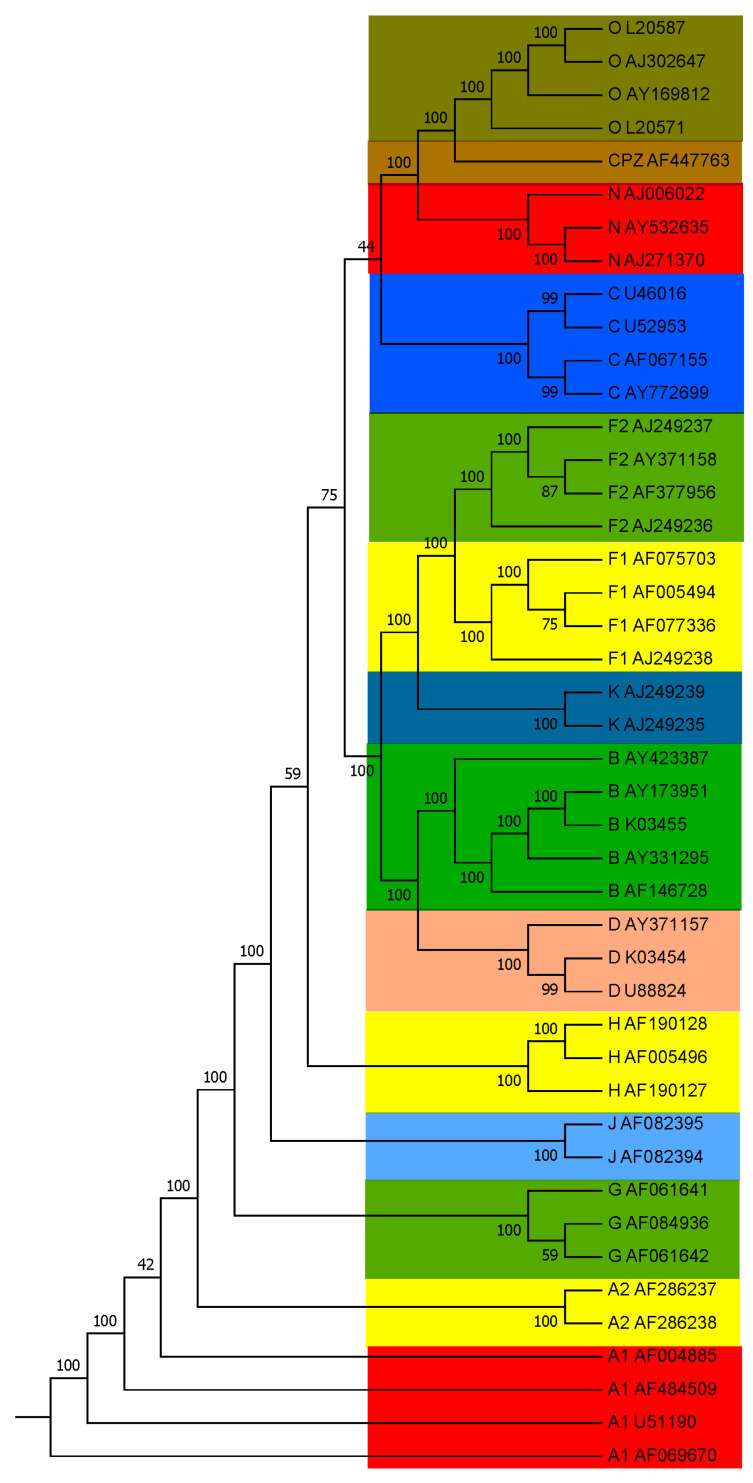
The modified bootstrap consensus tree for Figure 2 based on 100 replicates.

The modified bootstrap consensus tree for 44 HIV complete genome sequences is shown in Figure 5. As compared with Figure 2, the division of all HIV-1 genomes into Groups M, N, O, and CPZ is 100% supported. In Group M, each subtype branch is also 100% supported. In particular, in Subtype A and Subtype F of Group M, Sub-subtypes F1 and F2 (A1 and A2) are all 100% supported by the PWkmers. The branch of Subtype B and Subtype D is also supported by 100%. In Figure 2, Subtype C is divided into Group M, but in the consistent tree, as shown in Figure 5, Subtype C is divided out of Group M with a low supporting rate (44%).

We also compared the computational time required for our method in comparison to ClustalX [32] and DLTree [12]. On a modest PC (3.6 GHz quad core Intel Xeon processor, 4 GB RAM), for the whole genome sequences used in Table 1, it took 85 mins 54 secs for the alignment in ClustalX [32]. The DLTree model approach, which is a free-alignment method, used 20.3 secs of CPU time to get the distance matrices while the present *PWkmers* method only needs 5.8 secs of CPU time to get the distance matrices. This clearly shows the applicability of the *PWkmers* method for large datasets.

We also tested our method on three larger datasets: 867 HIV genomic sequences [5], 1625 HIV circulating recombinant form (CRF) genomic sequences, and 5596 pure subtype HIV genomic sequences from http://www.hiv.lanl.gov/ for k=8, respectively. We put these three datasets on https://github.com/myl446/HivStudy. Our method on our PC only takes 70secs, 244secs, and 46mins 52 secs for each dataset, respectively. For the two datasets including 867 HIV genomic sequences and 5596 pure subtype HIV genomic sequences, all HIV-1 sequences from the same subtype are clustered together with 100% accuracy, while for the dataset including 1625 HIV CRF genomic sequences, the accuracy is 88.35%.

### 3.2. Application of Our Method on Other Datasets

We also used another benchmark dataset including 48 complete genome sequences used in previously published papers [7,8] to evaluate our *PWkmers* method. All these sequences can be downloaded from NCBI (https://www.ncbi.nlm.nih.gov/). Details of these sequences can be found in [7,8]. Hepatitis E is an inflammation of the liver caused by infection by the HEV (hepatitis E viruses). Hepatitis E is divided into four genotypes, and classification is based on the nucleotide sequences of the complete genome. Genotype 1 has been classified into five subtypes, Genotype 2 into two subtypes, and Genotypes 3 and 4 into ten and seven subtypes [33], respectively.

The tree constructed by our *PWkmers* method (not shown here) indicates that 48 HEV genomes are grouped into four branches. Genotype 1 includes Subtypes Ia, Ib, Ic, Id, and Ie. Genotype 2 contains only a complete HEV genome M1. Genotype 3 includes Subtypes IIIa, IIIb, and IIIc. Genotype 4 includes Subtypes IVa, IVb, and IVc. This shows that our results are consistent with the accepted trees [34,35] and the reference tree constructed by ClustalX.

On the HEV dataset, we also compared the computational time of our method with ClustalX [32] and DLTree [12]. For the whole genome used in 48 HEV sequences, it took 87 mins 34 secs on our computer for alignment in ClustalX [32]. The DLTree model approach used 25.7 secs of CPU time to get the distance matrices while the present *PWkmers* method only needs 6 seconds of CPU time to get the distance matrices.

## 4. Discussion

Subtype classification has always been a focus in the field of virology, especially in the classification of HIV-1 viruses. Because of the wide range of viruses, sequence diversity, and rapid evolution, the development of HIV-1 vaccines is facing enormous challenges. In this work, we propose a new method to solve the problem of HIV-1 classification.

In our *PWkmer* method, we combined the number and position distribution of *k*-mers, and sequence length to capture more sequence information than traditional methods. In fact, our method records the average position of *k*-mers on the sequence. Ding et al. [36] presented an alignment-free method based on the normalized *k*-mers average interval distance to capture evolutionary information for sequence comparison. They only extracted the number and position distribution of *k*-mers. Tang et al. [37] presented the normalized *k*-mers average relative distance to improve the method of Ding et al. [36]. Nevertheless, in their methods, the determination of the *k* value requires empirical calculation, while we directly determine k=8 by score(k).

We computed the Robinson–Foulds distances between the phylogenetic trees reconstructed for different *k* by our method and the reference tree reconstructed by ClustalX on our HIV-1 dataset, which are shown in Table 2. It can be seen from Table 2 that when k=8, the Robinson–Foulds distances decrease to a lower value, which means that, with the further increase of *k*, the trees of HIV become unstable and its topological structures change little. From Figure 1 and Table 2, we can see that the relative change in the score value and the Robinson–Foulds distance is the same, which further implies the rationality of the score value defined by us. Furthermore, when k=8, the distance between the tree constructed by the *PWkmers* method and that constructed by ClustalX is the minimum. Therefore, in the subtyping of HIV-1 viruses, we recommend the *k* value of the string length to be 8.

The HIV subtype classification method based on sequence comparison mainly relies on three gene coding proteins: gag, pol, and env. There are controversies about the spread and origin of $SIV_{CPZ}$. In this study, as can be seen from Figure 2, $SIV_{CPZ}$ is more closely related to group O, and after the bootstrap test, it has a 100% support rate, which is consistent with the classification results based on the proteins env and pol in the HIV database (http://www.hiv.lanl.gov/). However, in the HIV database, the classification results based on the protein gag are consistent with the classification results of ClustalX, and $SIV_{CPZ}$ is classified outside Groups N and O. As compared with the benchmark dataset used in many studies [5,7], we added a sequence (AF146728, Subtype B, HIV-1 isolated from Australia) which was obviously misclassified by Chang et al. [8]. In our method, we correctly grouped it in Subtype B and the cluster was 100% supported in the bootstrap test. In Pandit et al. [16], the authors concatenated the first and last of 10 sequences in the same subtype, and then classified them according to the fractal dimension. However, given a new sequence, this method cannot be used to determine which subtype it is attached to, or to which subtype it belongs. On the other hand, our method can directly calculate to determine which subtype or sub-subtype the new sequence belongs to. Our results show that the *PWkmer* method is useful and efficient.

## 5. Conclusions

The subtype classification of species in virology has always been a challenging problem. With the development of sequencing technology, more and more complete genome sequences become available. However, traditional sequence alignment tools and evolutionary models are not efficient in dealing with large-scale genome sequences. In this study, we proposed a new method to solve the problem of the subtype classification of HIV-1. Validation of the Robinson–Foulds distance method and the modified bootstrap method shows that the presented method is reliable for the phylogenetic analysis of HIV-1. At present, the common method for virus subtype classification is based on multi-sequence alignment. Compared with multi-sequence alignment, our method is fast and accurate, and can process large-scale data.

The selection of the *k* value is very important. Specifically, if the *k* value is too small, *k*-mers cannot capture the tiny differences in the genome of different strains; if the *k* value is too large, it takes too much time and computer memory space for function *f* of all *k*-mers. To determine the optimal *k* value, we proposed a new method, which provides a quantitative index for its determination. We then found that the *k* value is independent of the number of genome sequences in the dataset. In summary, our method can capture the *k*-mer distribution information and provide a fast tool for whole genome sequence comparison analysis. We hope that our method will be useful in the phylogenetic analysis of within-species variants using their complete genome sequences.

## Figures and Tables

**Table 1:** Labels of complete genome builds used for 44 HIV-1 genomes of the dataset.

No.	Accession	Subtype	Length (bp)	Area
1	U51190	A1	8999	Uganda
2	AF004885	A1	9160	Kenya
3	AF069670	A1	8813	Somalia
4	AF484509	A1	8807	Uganda
5	AF286237	A2	9060	Cyprus
6	AF286238	A2	8972	DRC
7	AY173951	B	8996	Thailand
8	AY331295	B	8834	USA
9	AY423387	B	9359	Netherlands
10	K03455	B	9719	France
11	AF146728	B	8887	Australia
12	AF067155	C	9002	India
13	AY772699	C	9011	South Africa
14	U46016	C	9031	Ethopia
15	U52953	C	8959	Brazil
16	AY371157	D	8379	Cameroon
17	K03454	D	9176	DRC
18	U88824	D	8952	Uganda
19	AF005494	F1	8968	Brazil
20	AF075703	F1	8925	Finland
21	AF077336	F1	8903	Belgium (DRC)
22	AJ249238	F1	8614	France
23	AF377956	F2	8782	Cameroon
24	AJ249236	F2	8555	Cameroon
25	AJ249237	F2	8589	Cameroon
26	AY371158	F2	8349	Cameroon
27	AF061641	G	9047	Finland(Kenya)
28	AF061642	G	9074	Sweden (DRC)
29	AF084936	G	9707	Belgium (DRC)
30	AF005496	H	8953	Cent.Afr. Rep
31	AF190127	H	9056	Belgium
32	AF190128	H	9707	Belgium
33	AF082394	J	8943	Sweden
34	AF082395	J	8953	Sweden
35	AJ249235	K	8600	DRC
36	AJ249239	K	8604	Cameroon
37	AJ006022	N	9182	Cameroon
38	AJ271370	N	9045	Cameroon
39	AY532635	N	8938	Cameroon
40	AJ302647	O	9829	Senegal
41	AY169812	O	9110	Cameroon
42	L20571	O	9793	Cameroon
43	L20587	O	9754	Cameroon
44	AF447763	CPZ	9326	Tanzania

DRC: Democratic republic of Congo

**Table 2:** Robinson–Foulds distances between phylogenetic trees reconstructed by our method at k=2,3,…,9,10 in Manhattan distance and the tree reconstructed by ClustalX on the HIV dataset.

Species	*k* = 2	*k* = 3	*k* = 4	*k* = 5	*k* = 6	*k* = 7	*k* = 8	*k* = 9	*k* = 10
HIV	74	54	38	26	20	14	**10**	12	14

## Supplementary Materials

The following are available online at https://www.mdpi.com/1099-4300/22/2/255/s1.
