# Supplementary material for: Phylogenetic Analysis of HIV-1 Genomes Based on the Position-Weighted K-mers Method
Source: Entropy (Basel). 2020 Feb 23;22(2):255. doi: 10.3390/e22020255 (PMC7516702; doi:10.3390/e22020255)
Supplement: Supplementary file 1 [file entropy-22-00255-s001.pdf]

HivStudy

This is a method to study the phylogenetic relationships among complete genome sequences of different subtypes of viruses. The source codes of our method and datasets used can be downloaded from https://github.com/myl446/HivStudy

ComputerdistanceMatrix.java:This is a program for calculating the distance matrix of species. 5596data.rar : This dataset contains 5596 pure subtype HIV sequences.

datacg1625.rar : This dataset contains 1625 Circulating Recombinant Form (CRF) HIV sequences.

PureSequences867.rar: This dataset contains 867 pure subtype HIV sequences.

This program needs to install JDK 1.7, eclipse-jee-mars-2-win32-x86_64 to run.
